# Nutritional management of a patient with obesity and pulmonary embolism: a case report

**DOI:** 10.1186/s12937-016-0202-9

**Published:** 2016-10-19

**Authors:** Maria Luisa Fonte, Lauren Fietchner, Matteo Manuelli, Hellas Cena

**Affiliations:** 1 Department of Public Health, Experimental and Forensic Medicine, Unit of Human Nutrition, University of Pavia, Via Bassi, 21, 27100 Pavia, PV Italy; 2 Department of Pediatrics, Division of Pediatrics GI and General Academic Pediatrics Mass General Hospital for Children, Boston, Massachussets USA

**Keywords:** Obesity, Pulmonary embolism, Nutrition, Vitamin K, Nutritional assessment, Diet

## Abstract

**Background:**

The aim of this case report is to discuss the issue of nutritional therapy in patients taking warfarin. Patients are often prescribed vitamin K free diets without nutritional counseling, leading to possible health consequences.

**Case presentation:**

A 52-year-old woman with obesity and hypertension was prescribed a low calorie diet by her family doctor in an effort to promote weight loss. After a pulmonary embolism, she was placed on anticoagulant therapy and on hospital discharge she was prescribed a vitamin K free diet to avoid interactions. Given poor control of her anticoagulant therapy, she was referred to our Nutritional Unit outpatients’ service.

**Conclusions:**

This case illustrates the importance of a thorough medical nutrition assessment in the management of patients with obesity and the need for a change in the dietary approach of nutritional therapy in the management of vitamin K anticoagulant therapy. In patients taking warfarin, evidence suggest that the aim of nutritional therapy should be to keep dietary intake of vitamin K constant.

## Background

A number of studies have found a significant increased risk for deep venous thromboembolism (VTE), and/or pulmonary embolism in people with obesity [1–6]. A hazard ratio of 2.7 for a body mass index >40 has been reported by the Atherosclerosis Risk in Communities (ARIC) and the Cardiovascular Health Study (CHS) [2].

Besides obesity, many other factors contribute to increasing the VTE risk: smoking, increasing age, patients with factor V Leiden or pro-thrombin gene mutation [7] and oral contraceptive therapy [8].

Patients who had a VTE episode are usually treated with oral anticoagulants. Their use is challenging because the therapeutic range is narrow and dosing is affected by multiple factors including genetic variation, drug interactions, and diet [9]. Moreover, compared to normal weight, obese and morbidly obese patients had a decreased initial response to warfarin [10, 11].

Prothrombin time is an assay evaluating the extrinsic pathway of coagulation. The international normalized ratio (INR) is used to standardize the prothrombin time and the optimal intensity of anticoagulant therapy corresponds to a target INR of 2.5 (range, 2.0 to 3.0) [12].

Both prophylaxis and treatment of VTE and pulmonary embolism (PE) with anticoagulants are associated with significant risks of major and fatal hemorrhage. Both anticoagulant treatment and lifestyle changes should be individualized to prevent further complications [9, 12].

Two major factors that may lead to poor INR control are failure to take warfarin appropriately and very low dietary vitamin K intake/vitamin K deficiency. The nutritional management of these patients is therefore extremely complicated.

We hereby describe a case of pulmonary embolism in an obese patient providing key insights into treatment with medical nutrition therapy and anticoagulants.

## Case presentation

### In-patient diagnosis and treatment

In January 2015, a 52-year old woman with obesity and hypertension was admitted to the cardiac intensive care unit of a hospital in Northern Italy for acute dyspnea and chest pain. Computed tomography angiography revealed filling defects affecting the right and left branches of the pulmonary artery suggestive of pulmonary embolism (PE). She was initially treated with intravenous heparin sodium and bridged to warfarin as a long-term anticoagulant. Before the hospital discharge she was prescribed a vitamin K free diet reported in Table 1(b). Although the general recommendation is to maintain a stable and consistent intake of Vitamin K, in the clinical practice patients are suggested to avoid food high in vitamin K and they are given general dietetic guidelines.

**Table 1:** Total energy and nutrient composition of three diets prescribed to a 52-year-old woman before (a) and after (b, c) a pulmonary embolism

	(a) Low calorie diet		(b) vitamin K free diet		(c) NU diet	
Nutrients	Amount (units)	% of total energy	Amount (units)	% of total energy	Amount (Units)	% of total energy
Energy	1044 (kcal)		922 (kcal)		1688 (kcal)	
Proteins	50 (g)	19	30 (g)	13	77 (g)	18
Animal proteins	34 (g)		17 (g)		46 (g)	
Vegetable proteins	16 (g)		13 (g)		31 (g)	
Fats	37 (g)	32	37 (g)	36	50 (g)	26
Animal fats	13 (g)		32 (g)		12 (g)	
Vegetable fats	24 (g)		5 (g)		38 (g)	
Saturated fats	11 (g)	9.5	18 (g)	17	11 (g)	6.0
Monounsaturated fats	20 (g)	17	10 (g)	10	30 (g)	16.0
Linoleic acid	3 (g)	2.5	3.0 (g)	3.0	6 (g)	3.0
Linolenic acid	1 (g)	1.0	1.0 (g)	1.0	1 (g)	0.5
Polyunsaturated fats	4 (g)	3.5	4.0 (g)	4.0	8 (g)	4.0
Cholesterol	97 (mg)		103 (mg)		236 (mg)	
Carbohydrates	139 (g)	50	127 (g)	51	250 (g)	56
Soluble carbohydrates	42 (g)	15	41 (g)	17	52 (g)	11
Dietary fiber	16 (g)		14 (g)		23 (g)	
Thiamine	0.6 (mg)		1.1 (mg)		1.0 (g)	
Vitamin B6	1.5 (mg)		1.1 (mg)		1.9 (g)	
Folate	253 (μg)		87 (μg)		264 (g)	
Vitamin K	273 (μg)		23 (μg)		58 (μg)	
Sodium	1880 (mg)		3415 (mg)		1460 (g)	

a) Low calorie diet prescribed the patient by her family doctor before the pulmonary embolism. b) Vitamin K free diet prescribed upon discharge to the patient taking oral anticoagulants after the pulmonary embolism. c) Diet prescribed by the Nutritional Unit (NU) to the same patient: moderately low calorie with low vitamin K (planning a slow and continuous increase of vitamin K daily intake up to 150 μg per day)

Diet composition analyzed by trained dietitians using a dedicated software developed by our Nutrition Unit incorporating “Food Composition Database for Epidemiological Studies in Italy” by Gnagnarella P, Salvini S, Parpinel M. Version 1.2015 Website http://www.bda-ieo.it/

**Fig. 1:**
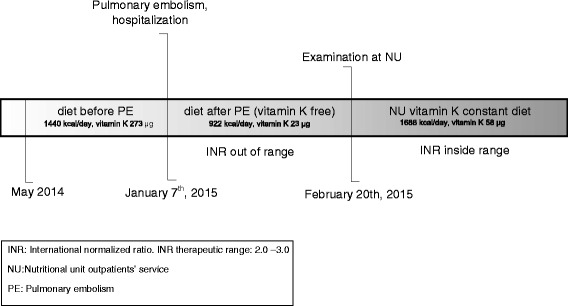
Timeline

During her first month as an outpatient it was very difficult to get her INR into the therapeutic range. Her family doctor recognized that poor nutrition is an important factor in establishing therapeutic levels and she was referred to our Nutritional Unit outpatient service (NU) for a nutritional assessment and specific medical nutrition therapy. Figure 1 shows the timeline of present illness, intervention and follow-up.

### Outpatient nutritional unit

#### Medical history

She reported a long-term history of obesity and hypertension. She denied allergies and cigarette smoking and reported a sedentary lifestyle with no physical activity. The patient was nulliparous and had been on oral contraceptive therapy since the age of 25. The therapy was discontinued by her physicians after her PE. At the time of the examination she was on nebivolol, alprazolam and warfarin adjusted for INR, as well as vitamin B12 and folate supplementation (400 μg). Her family history was negative for thrombosis and cardiovascular diseases.

#### Weight history

The patient reported that she began struggling with obesity during her childhood. She began adulthood at a weight of 90 kg (BMI 35.0 kg/m^2^). Maximum and minimum weight reported by the patient were 117.5 kg (BMI 45.9 kg/m^2^) and 69 kg (BMI 27.0 kg/m^2^) respectively at 51 and 26 years of age. Soon after reaching her peak weight she started a hypocaloric diet as suggested by her family doctor, Table 1(a).

### Nutritional assessment

Anthropometrical, bioelectrical and clinical findings at physical examination are showed in Table 2 with biochemical analyses. The patient’s BMI was greater than 34.99 Kg/m^2^, class II obesity according to WHO classification [13]. Fat mass was estimated by bioelectrical impedance analysis (BIA) using a BIA 101 Akern s.r.l. (Italy). The exam, performed under standardized conditions [14] revealed an important increase in fat mass although a normal state of hydration. The waist-to-hip ratio indicates an android fat distribution. Evidence of increased visceral fat depot is shown by waist circumference measurement.

**Table 2:** Clinical findings and blood test results at the time of nutritional unit outpatient service examination

Blood test results	
Variables	Values (Units)
Hemoglobin	13.4 (g/dl)
Hematocrit	40.8 (%)
Mean cellular volume	83.2 (fl)
Platelets	250 (1000/ μl)
Leukocytes	8.81 (1000/ μl)
International normalized ratio	2.06
Antithrombin III actvity	99 (%)
D-dimer	378 (μg/l)
Anti-cardiolipin antibodies	negative
Anti-beta 2-glycoprotein I antibodies	negative
Homocysteine	21 (μmol/L)
Folate	5.8 (ng/ml)
Vitamin B12	372 (pg/ml)
Vitamin D	7.4 (ng/ml)
Parathormone	35.3 (pg/ml)
Glucose	88(mg/dl)
Insulin	18.6 (mIU/ml)
Homeostatic model assessment (HOMA) index	4.04
Magnesium	2 (mg/dl)
Prealbumin	0.23 (g/l)
Weight: 93.6 kg Height: 160 cm Body mass index: 36.6 kg/m^2^ ^a^Blood pressure: 120/70 mmHg Waist circumference: 122 cm Hip circumference: 118 cm Waist-to-Hip ratio: 1.03	^b^Resting Energy Expenditure (REE): 1515 kcal/day ^c^Energy Expenditure: 1818 kcal/day ^d^BIA for BC FM = 48.2 % PhA = 5.5°

VR506Q mutation of factor V (Leiden): absent

VH1299R mutation of factor V: absent

G20210 of protrhombin gene (factor II): absent

Methylene tetrahydrofolate reductase (MTHFR) gene mutation: absent

Fifty-two-year old female patient with obesity and hypertension taking anticoagulants due to a history of a recent pulmonary embolism

^a^taking hypertension medication

^b^Mifflin equation [14].

^c^EE = REE x Activity Factor Category Definition (1.2 for sedentary)

^d^Bioelectrical Impedance Analysis for Body Composition (BIA 101 Akern s.r.l. – Italy); FM = Fat mass, PhA = Phase angle

Resting energy expenditure (REE) has been estimated by means of Mifflin predictive equations [15], which is more likely than the other known equations to estimate REE to within 10 % [16].

Before the PE, the patient was following the diet reported in Table 1(a), which was prescribed by her family doctor. Total energy and nutrient intakes were analyzed by trained dietitians using dedicated software developed by our Nutrition Unit incorporating “Food Composition Database for Epidemiological Studies in Italy” by Gnagnarella P, Salvini S, Parpinel M. (Version 1.2015 Website http://www.bda-ieo.it/).

The diet followed by the patient before the PE was a low-calorie diet that did not satisfy the dietary recommendations for protein and micronutrient intake (LARN 2014) [17].

The nutrient composition of the diet prescribed to the patient after the PE upon hospital discharge is reported in Table 1(b).

Vitamin K free diets typically are lacking in vegetables, with a consequent insufficient dietary fiber and folate intake. Protein intake prescribed to the patient was far lower than any standard recommendation (less than 0.33 g/ kg of body weight) while the total fat and in particular the saturated ones were higher than the standard recommendations (LARN 2014 and ESC 2012 [18]).

The patient’s laboratory values at the time of her first examination in our NU are shown in Table 2. She had high levels of homocysteine and low prealbumin. She also was vitamin D deficient as can be seen in patients with obesity [19].

Both folate and vitamin B12 concentrations levels were within the accepted normal ranges, likely due to her supplementation and not her dietary intake.

### Therapeutic intervention

After the nutritional assessment, a balanced, low-calorie diet was prescribed, with particular attention to sodium and soluble carbohydrates intake. The protein intake prescribed was equivalent to 0.8 g/Kg of actual body weight. The nutritional composition of the diet prescribed at our NU is shown in Table 1(c).

In order to avoid a sharp change in vitamin K intake the starting vitamin K intake prescribed was low (57.88 μg per day). Gradually her vitamin K intake was increased to 150 μg per day. Folate supplementation was prescribed (400 μg per day) as well as vitamin D supplementation (300,000 IU every 3 months for 9 months) [20].

The benefit of having her seen in the NU was that our staff was able to take the appropriate amount of time to counsel the patient and to ensure she understood the dietary guidelines and risk of complications in order to transform the acquired knowledge into behavior change and to achieve greater adherence.

### Follow-up and outcomes

The patient’s INR values were tested on a regular base. As shown in Table 3, the INR values were therapeutic and remained constant immediately following initiation of the prescribed diet.

**Table 3:** INR testing in a 52-year old woman taking oral anticoagulants due to a recent history of pulmonary embolism

Date (2015)	Weekly warfarin dose (mg)	International normalized ratio
01/19	41.75	1.82
01/26	43.75	1.80
02/02	45	3.16
02/09	43.75	1.82
02/20	43.75	2.06
02/27	41.75	2.23
03/13	41.75	2.16
03/20	43.75	2.43
04/03	43.75	2.04

Before February 20th, she was fallowing a vitamin K free diet. After February 20th, she was following the diet prescribed at our nutritional unit (NU), with stable content of vitamin K

INR = International normalized ratio. INR therapeutic range: 2.0 – 3.0

Warfarin dose refers to the week prior to the INR value

## Conclusion

This case study presents the nutritional issues of anticoagulants in the setting of obesity. We present a case of a 52 year old female patient with obesity and hypertension taking anticoagulants due to a history of a recent PE treated in our NU outpatient service.

She had no inherited thrombophilia nor a previous hospitalization, immobility, trauma, surgery, malignancy or infection before the PE, and we hypothesize that perhaps this adverse event might have been caused by the combination of obesity, oral contraceptive therapy and sedentary lifestyle. Indeed, there is strong evidence that oral contraceptives entail a statistically significant risk of VTE, which is particularly high among women with high body mass indices and a history of cigarette smoking [21, 22]. Previous studies demonstrate that oral contraceptives modify the effect of obesity on the risk of venous thrombosis, with a 10-fold increased risk among women on oral contraceptives with a BMI >25 kg/m^2^ compared to normal weight women not using oral contraceptives [8, 23].

Obesity is considered a risk factor for VTE and PE and is associated with an increase of procoagulant factors (factor VII, factor VIII, factor XII and fibrinogen) [24–26] and with venous stasis [2] in turn increasing the thrombotic risk (Virchow’s triad). Analysis of data from the National Hospital Discharge Survey indicated an increased relative risk for VTE (RR 2.50, 95 CI 2.492.51) and PE (RR 2.21, 95 % CI 2.202.23) in subjects with obesity [27].

Our patient reported a long history of obesity and 7 months before the PE episode she was put on a low calorie diet without a formal nutritional assessment of her specific nutritional needs.

As shown in Table 1, the diet before the PE did not satisfy fiber, protein and micronutrients needs for this patient likely due to the low fruit and vegetable intake.

The “Longitudinal Investigation of Thromboembolism Etiology (LITE)” prospective study [28] found a diet with more fruits, vegetables, and fish, and less red and processed meat was associated with a lower VTE incidence. Data about the relationship of diet to VTE risk comes from: historical observations about the incidence of PE under wartime conditions, including food rationing in early 20th century European cities; prospective observational studies of diet and lifestyle factors associated with VTE [9]; case–control studies of VTE patients looking at lipid profiles, inflammation markers, and coagulation variables; comparisons among the general population on various diets regarding lipid profiles, inflammation markers, and coagulation variables [9]. It is possible that the link between diet and VTE is potentially through other aspects of their lifestyle: people who are less health conscious may be less concerned about a healthy diet and may place less importance as well on physical activity, exposing them to a greater risk of prolonged non-pulsatile venous blood flow and consequent valve pocket hypoxemia. According to the valve cusp hypoxia hypothesis (VCHH) deep venous thromboembolism may occur wherever sustained non-pulsatile venous blood flow leads to suffocating hypoxemia in the valve pockets, resulting in hypoxic injury to and hence death of the inner endothelium of the cusp leaflets [9].

Furthermore both diets before and after the PE were very high in sodium intake, which would be inappropriate in a patient with a long history of hypertension. Our patient also had hyperhomocysteinemia, despite vitamin B12 and folate supplementation. High plasma concentration of homocysteine may be due to genetic defects in the enzymes involved in homocysteine metabolism (i.e. homozygosity for the thermolabile variant of methylene tetrahydrofolate reductase, MTHFR -TT genotype), nutritional deficiencies in vitamin cofactors (folate, vitamin B6, and vitamin B12 [29–32]), or other factors including some drugs such as oral contraceptives. Given her absence of MTHFR mutation, it is most likely her hyperhomocysteinemia was due to both the long-term oral contraceptive therapy and to a low folate diet. Since prothrombotic effects of homocysteine have been demonstrated [33–36], it is important to include this parameter in the nutritional assessment. Indeed, a proper assessment of patients with obesity should include vitamin status especially in those at risk of subclinical deficiencies.

This case illustrates the need for a thorough medical nutrition assessment in the management of patients with obesity. Weight loss therapy, cannot be focused only on lowering calories, but should include care to meet macro and micronutrients recommendations even through supplementation, if necessary.

A vitamin K free diet, such as Table 1(b), is very often prescribed to patients who had a PE and are on warfarin. Individuals on warfarin generally are sensitive to fluctuations in vitamin K intake, and adequate INR control requires close attention to the amount of vitamin K ingested from dietary and other sources [37]. This case report highlights the need for a change in the clinical approach of patients taking warfarin. The aim of nutritional therapy should be to keep dietary intake of vitamin K constant rather than to exclude it completely from the diet, which is not uncommon in the clinical practice. Several studies have demonstrated the relationship between vitamin K intake and INR control [38–40] in particular suggesting patients should be advised to consume a healthy diet, and they should not avoid fruits and vegetables for fear of altering the INR.

Despite the evidence, it is common practice to suggest vitamin K free diets to all patients on oral anticoagulants therapy. The main consequence of this recommendation is the exclusion of vegetables and fruit from the diet, resulting in nutrient deficiencies and impaired health status. We should not forget that eliminating these very important food groups puts our patients at further risk.

In the field of cardiovascular risk and diseases, medical nutrition therapy plays a crucial role in providing both prevention and therapy. A correct and complete nutritional assessment is essential for effective therapy. Nutritional intervention should be tailored to the individual patient and should provide the correct amount of macronutrients, micronutrients and vitamin or mineral supplementation if needed.

## Abbreviations

ARIC: Atherosclerosis Risk in Communities
BIA: Bioelectrical impedance analysis
BMI: Body mass index
CHS: Cardiovascular Health Study
INR: International Normalized Ratio
LITE: Longitudinal Investigation of Thromboembolism Etiology
MTHFR: Methylene tetrahydrofolate reductase
NU: Nutritional Unit outpatient service
PE: Pulmonary embolism
REE: Resting energy expenditure
VCHH: Valve cusp hypoxia hypothesis
VTE: Venous thromboembolism
WHO: World Health Organization
