# Erratum to: Nutritional management of a patient with obesity and pulmonary embolism: a case report

**DOI:** 10.1186/s12937-017-0231-z

**Published:** 2017-02-01

**Authors:** Marria Luisa Fonte, Lauren Fiechner, Matteo Manuelli, Hellas Cena

**Affiliations:** 1 Department of Public Health, Experimental and Forensic Medicine, Unit of Human Nutrition, University of Pavia, Via Bassi, 21, 27100 Pavia, PV Italy; 2 Department of Pediatrics, Division of Pediatrics GI and General Academic Pediatrics Mass General Hospital for Children, Boston, Massachussets USA

## Erratum

After the publication of this article [1] it was noticed that there was a misspelling in the author name – Lauren Fiechner where an extra ‘t’ was added.

The original article was corrected.

The publisher apologises for this error.
